# Safety and efficacy of DLL3 CAR-T cells armored with dnTGFBR2 in a subject with recurrent small-cell lung cancer: case report

**DOI:** 10.3389/fonc.2026.1885732

**Published:** 2026-07-07

**Authors:** Chelsey M. Williams, Susanne M. Arnold, John L. Villano, Erika N. Amarilla Alonso, Christine F. Brainson, Paul O. Schwarzenberger, Sahista Vahora, Ayman Qasrawi, Reinhold Munker, B. Mark Evers, Zhonglin Hao

**Affiliations:** 1 Department of Internal Medicine, Division of Medical Oncology, University of Kentucky Markey Cancer Center, Lexington, KY, United States; 2 University of Kentucky College of Medicine, Lexington, KY, United States; 3 Department of Toxicology and Cancer Biology, University of Kentucky Markey Cancer Center, Lexington, KY, United States; 4 Legend Biotech USA Inc., Somerset, NJ, United States; 5 Department of Medicine, Division of Hematology and Cellular Therapy, University of Kentucky Markey Cancer Center, Lexington, KY, United States; 6 Department of Surgery, Markey Cancer Center, University of Kentucky Markey Cancer Center, Lexington, KY, United States

**Keywords:** CAR-T, case report, DLL3, ES-SCLC, LB2102

## Abstract

Novel CAR-T therapies targeting delta-like ligand 3 (DLL-3) are promising treatment strategies for individuals with extensive-stage SCLC (ES-SCLC). We report a case of a patient with ES-SCLC who received investigational therapy LB2102 off-label after becoming ineligible for a clinical trial. We described her positive, long-term response to treatment. This case highlights efficacious use of this investigational drug in a real-world setting prior to release of final reported data from phase 1 trial NCT05680922.

## Introduction

ES-SCLC carries a poor prognosis and often involves the central nervous system (CNS). Existing systemic treatment options include chemotherapy, immunotherapy and radiation (1). Recently, novel precision medicine-based approaches that target cell surface protein DLL3 have been developed. DLL3 is an inhibitory notch ligand that is highly expressed in SCLC (2). Although DLL3 is highly expressed by SCLC cells, expression in normal tissue is low, therefore strategies to target DLL3 have minimal toxicity (3, 4). LB2102 is an investigational, autologous DLL3-targeted CAR-T cell product with dominant-negative transforming growth factor beta receptor type 2 (TGFBR2) armor (4). Genetic modification of endogenous TGFBR2 on CAR-T cells allows LB2102 to target DLL3-positive SCLC while overcoming the immunosuppressive tumor microenvironment. A multicenter, open-label phase 1 trial evaluating LB2102 in patients with SCLC and large cell neuroendocrine carcinoma (LCNEC) who have relapsed disease or are refractory to prior lines of therapy is ongoing (LB2102–100, NCT05680922). In this report, we describe our patient’s positive response to a single infusion of LB2102 administered under a single patient investigational new drug (IND) procedure after she became ineligible for continuation in the phase 1 trial.

## Case presentation

The patient is a 51-year-old female diagnosed with ES-SCLC who presented with a central mediastinal mass, liver metastases, and bony metastases (**Figure 1**). She also had a T9 pathologic compression fracture with debilitating cancer-related pain. She received 5 cycles of cisplatin, etoposide, and atezolizumab followed by maintenance atezolizumab. Within 10 months after completion of initial chemotherapy, she developed persistent headache with disequilibrium. Magnetic resonance imaging (MRI) of the brain revealed metastases, which were treated with whole-brain radiation. She was then enrolled in the LB2102–100 study, at a planned dose of 2.0×10^6^ T-cells/kg body weight. She received bridging chemotherapy with irinotecan D1 and D8 every three weeks each cycle for 2 cycles. One month later she developed acute encephalopathy requiring hospitalization. Lumbar puncture with cerebrospinal fluid studies were unrevealing. Electroencephalogram showed diffuse slowing consistent with encephalopathy. MRI brain demonstrated stable metastatic frontal lesion. Her symptoms were ultimately attributed to delirium secondary to use of extended-release morphine prescribed to manage cancer-related pain. There were concerns regarding her compliance with pain medications, and per sponsor guidance, she was deemed ineligible to continue in the LB2102 study. With agreement from the manufacturer of LB2102 (Legend Biotech), a single-subject emergency IND application for LB2102 therapy was filed and approved by the Food and Drug Administration. Enrollment criteria of the single-subject IND were modified to allow CAR-T treatment. She underwent lymphodepletion with fludarabine 30 mg/m² and cyclophosphamide 300 mg/m². Baseline immunohistochemistry analysis showed that 88% of her tumor biopsy specimen had an expression score for DLL3 ≥2+, indicative of high tumor expression. She tolerated infusion of LB2102 (2.0 x 10^6^ CAR-T cells/kg) without difficulty. She did not experience any dose-limiting toxicity, cytokine release syndrome (CRS), or immune effector cell-associated neurotoxicity (ICANS). Four weeks later she remained anemic (grade 2) and neutropenic (grade 4). Cytopenias recovered without intervention by week 10 following treatment and no growth factor support needed. C-reactive protein (CRP), ferritin, lactate dehydrogenase (LDH), and gamma-glutamyl transferase (GGT) peaked 11 days after LB2102 infusion. Maximum CRP value was 160 mg/L (<3), ferritin was 829 mg/ML (13–150), LDH was 307 U/L (115-250), and GGT was 141 U/L (5-36).

**Figure 1:**
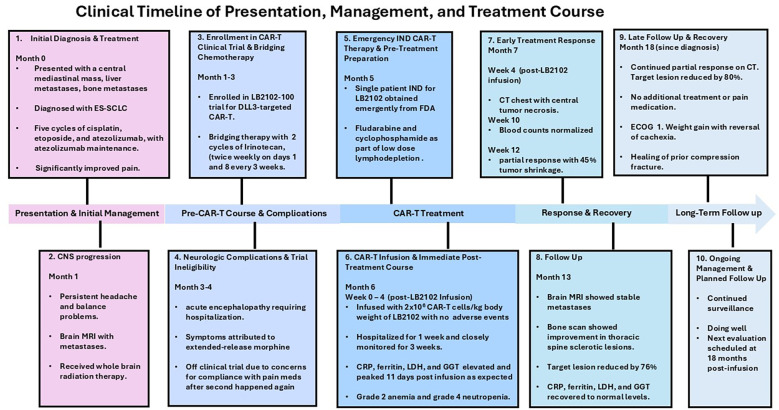
Timeline describing patient’s clinical presentation, treatment events, and long-term follow-up.

Repeat computed tomography (CT) of the chest, abdomen, and pelvis revealed central tumor necrosis 4 weeks after LB2102 infusion. By week 12, the target lesion had shrunk by 35%, consistent with partial response per response evaluation criteria in solid tumors (RECIST) 1.1 (**Table 1**). MRI of the brain performed 5 months after LB2102 infusion showed stable metastatic disease with post-treatment changes in white matter. Nuclear medicine bone scan demonstrated improvement in thoracic spine lesions. No liver lesions were visualized on post-treatment CT scan. Peripheral CAR-T cell count was 2710/mL, with 0.86% CD3+ T cells, 0 circulating tumor cells (CTC, from 27/mL at baseline), 0 DLL3+ CTCs (from 6/mL at baseline), and 160 CD4+ T cells/mL. Previous elevations in levels of CRP, ferritin, LDH, and GGT normalized (see reference range above). At 1 year, she has not required any further cancer treatment and has maintained an eastern cooperative oncology group (ECOG) performance status of 1. Her cancer-related pain and cachexia have resolved. Repeat CT chest imaging shows no cancer progression after 1 year. Total target lesion reduction is 100% (**Figure 2**) with healing compression fractures and no pain medications requirement.

**Table 1:** RECIST1.1 measurement for VL (sIND).

Lesion location (mm)	8/29/2024 baseline	9/27/24 week 4	11/26/2024 week 12	2/28/2025 6 months	8/29/2025 12 months
TL1 (medias)	31	20	15	13	0
Change TL(%)		-35	-52	-58	-100
Response		PR	PR	PR	CR
NT1 (right upper paratracheal)	12	13	6	4	0
NT2	+	+	+	+	+
NT3	+	+	+	+	+
NT4 (R. A)	+	+	+	+	+
New lesion	n	n	n	n	n
Disease State					
RECIST 1.1		PR	PR	PR	PR

**Figure 2:**
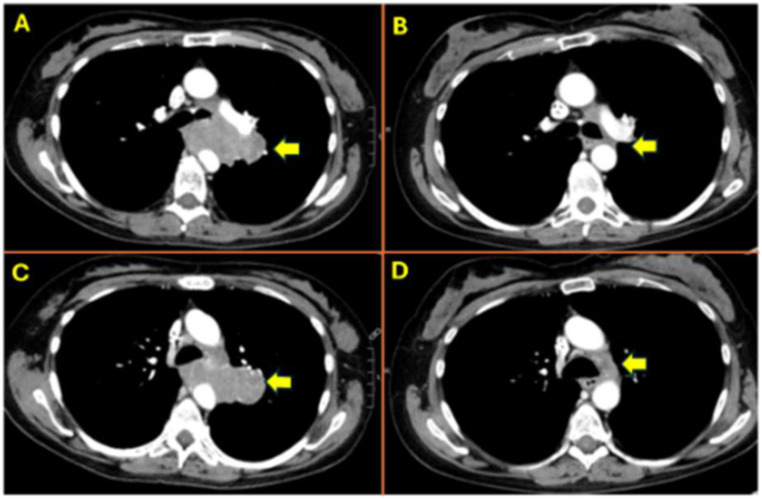
Contrast CT images before [**(A, C)** baseline] and 12 months after **(B, D)** infusion of LB2102. CT slice 5mm. Mass change in size in one year (yellow arrow). Partial response per RECIST 1.1.

## Discussion

This patient was enrolled in a phase I protocol (NCT05680922; A First in Human Dose Escalation and Cohort Expansion Study of DLL3-directed Chimeric Antigen Receptor T-cells in Subjects with Extensive Stage Small Cell Lung Cancer) before deemed ineligible. The protocol stipulates that “serious and/or uncontrolled medical condition that, in the Investigator’s judgment, would cause unacceptable safety risk, interfere with study procedures or results, or compromise compliance with the protocol” would be excluded. The sponsor strongly feels that it is not safe for her to participate due to non-compliance with the pain medication recommendation after two episodes of morphine caused delirium. The investigator feels however, with patient and family education and monitoring, LB2102 can be safely administered without compromise of safety given the generally benign safety profile of DLL3 targeted drugs, hence pursued sIND which the sponsor generously agreed to support.

In this subject, a one-time infusion of LB2102 was associated with a durable partial response and control of CNS disease. She did not experience CRS or ICANS following LB2102 infusion despite prior brain metastases and radiation therapy. Clinical interpretation of her CRP, ferritin, LDH, GGT, and CAR-T cell count indicates that she experienced minimal toxicity in response to treatment with expected expansion of CAR-T cells.

Tumors secrete TGF-β, which binds to TGFBR2 on T-cells to shut down their cancer-killing activity. With dnTGFBR2, engineered T cells do not respond to TGF-β allowing them to remain active in the tumor microenvironment. Despite the armor, no CRS or ICAN was observed. Indeed, some preliminary data indicate she had the best/highest CD4/CD8 ratios which might have contributed to no DLT, nor CRS or ICAN.

The ongoing clinical trial of LB2102 (NCT05680922) assesses the feasibility of single-administration DLL3-directed therapy in relapsed or refractory SCLC and LCNEC. Strict criteria prevented our patient from continuing in the trial. Patients who do not qualify for trials may also benefit from investigational DLL3-directed therapies. This case represents a real-world example of a durable systemic response with stable intracranial disease to novel LB2102 therapy 18 months post whole brain radiation, despite her exclusion from the study.

DeLLphi-304 (5) reported the efficacy and safety of tarlatamab against chemotherapy with either topotecan or lurbinectedin or amrubicin in the second line treatment of SCLC after relapse. The OS of the 254 patients in the tarlatamab arm was significantly longer (13.6 months, 95% CI 11.1–NR, HR 0.60, range 0.47–0.77, P<0.001) than the 255 patients in the chemotherapy group (OS 8.3 months, 95% CI 7.0–10.2 months). Grade 3 or higher AEs were lower (54% vs 80%) in the tarlatamab arm. Discontinuation of treatment rate was lower compared to chemotherapy (5% vs 12%). Importantly, tarlatamab was well tolerated with the majority of patients reporting mild to moderate severity occurring rarely or occasionally on trial. Tarlatamb was effective controlling the CNS disease (6).

While additional DLL-3 therapies such as Tarlatamab therapy have also been shown to produce sustained clinical benefit, treatment requires bi-weekly intravenous injections. In contrast, LB2102 is administered in a single infusion, reducing strain on infusion clinic resources and patient travel to treatment centers.

Authors would like to point out the following limitations of this report: (1) This is a single case report that needs to be confirmed with the ongoing phase I study results; (2) patient compliance issues may confound results; (3) although no lack pharmacokinetic/pharmacodynamic data, immune cell profile showed later she had the highest CD4/CD8 ratio of all patients treated with LB2102 (data not shown); (4) follow up is still ongoing.

## Conclusion

Strict clinical trial criteria may inhibit patient continuation or enrollment in investigational studies for ES-SCLC patients. This case with LB2102 was a one-time CAR-T cell treatment for patients with ES-SCLC This case suggests potential safety and clinical activity of LB2102 in previously treated SCLC and supports further investigation in this population.

## Data Availability

The original contributions presented in the study are included in the article/supplementary material, further inquiries can be directed to the corresponding author/s.
